# Hey Google, Remind Me to Write in My Diary: Voice Assistants for Daily Pain Monitoring

**DOI:** 10.1093/geroni/igaa057.2723

**Published:** 2020-12-16

**Authors:** Marcia Shade, Rasila Soumana Hama, Kyle Rector, Kevin Kupzyk

**Affiliations:** 1 University of Nebraska Medical Center, Omaha, Nebraska, United States; 2 University of Iowa, Iowa city, Iowa, United States

## Abstract

Diaries can be important tools to document and communicate pain symptoms. Diary-based assessments can be prone to poor adherence and limitations with biased recall. One strategy to help adherence is to use voice assistant reminders. A sample of 15 community dwelling aging adults used the Google Assistant for reminders to complete pain self-management tasks. One task was a reminder created to write daily in a pain diary. Within the diary, participants could document a change in pain, pain severity, average and worst amount of pain, and pain relief. At follow-up, it was noted that participants adhered to writing most pain characteristics in the diaries, but there were times that each question was not answered. Participant’s pain characteristics varied, which could be a helpful assessment to communicate to health providers for pain management. This feasibility study is preliminary support that voice assistants may help with the daily pain diary completion.

